# The relationship between organizational support, professional quality of life, decent work, and professional well-being among nurses: a cross-sectional study

**DOI:** 10.1186/s12912-024-02114-5

**Published:** 2024-06-25

**Authors:** Jie Zheng, Shengya Feng, Rong Gao, Xiaoyan Gong, Xinyu Ji, Yuling Li, Xiangli Wang, Bowen Xue

**Affiliations:** 1 School of Nursing, Shanxi Medical University, Taiyuan, Shanxi 030607 China; 2 The First Hospital of Shanxi Medical University, Taiyuan, Shanxi 030001 China; 3 The Second Hospital of Shanxi Medical University, Taiyuan, Shanxi 030001 China; 4 Affiliated Mental Health Center & Hangzhou Seventh People’s Hospital, Zhejiang University School of Medicine, Hangzhou, Zhejiang 310007 China

**Keywords:** Organizational support, Professional quality of life, Decent work, Professional well-being, Mediation analysis

## Abstract

**Background:**

Nurses often face challenges such as inadequate welfare protection, injustice, and workplace adversity including violence, bullying, and sexual harassment. In this context, providing sufficient support to nurses is crucial for the promotion of their professional well-being. This study examines the direct and indirect effects of perceived organizational support on nurses’ well-being, particularly highlighting the mediating roles of professional quality of life and the perception of decent work.

**Methods:**

A cross-sectional survey design was employed in this study. Convenience sampling was used to survey 792 nurses from five tertiary A-grade hospitals in Shanxi Province in January 2024. Data collection tools included a custom demographic survey, the Perceived Organizational Support Scale, Professional Quality of Life Scale, Decent Work Perception Scale, and Nurse Occupational Well-being Questionnaire. Descriptive statistics, correlation analysis, and mediation effect analyses were performed.

**Results:**

The findings demonstrate that perceived organizational support has a direct impact on nurses’ occupational well-being (β = 0.323, *p* < 0.001). Additionally, professional quality of life and the perception of decent work play chain mediating roles between perceived organizational support and nurses’ well-being (*β* = 0.019, BootLLCI = 0.010, BootULCI = 0.030).

**Conclusions:**

This study highlighted the importance of organizational support in enhancing nurses’ well-being. Professional quality of life and decent work were key mediators. Healthcare institutions should prioritize support measures to improve nurses’ well-being. Future research should explore additional mediators and mechanisms to develop effective strategies for nursing policymakers and administrators.

**Supplementary Information:**

The online version contains supplementary material available at 10.1186/s12912-024-02114-5.

## Backgrounds

Healthcare professionals globally are confronting substantial challenges posed by the persistent prevalence of influenza, the COVID-19 pandemic, and potential future infectious disease epidemics. Studies have shown that these individuals are subject to considerable stress, anxiety, post-traumatic stress disorder (PTSD), and persistent risk of infection, culminating in significant physical and psychological burdens [1, 2]. Compounding these challenges, the millennial generation is increasingly central to healthcare delivery, yet they face unparalleled employment pressures amid economic recessions, workforce migrations, and the advent of artificial intelligence [3]. Moreover, the demanding healthcare environment, exacerbated by inadequate welfare protections, diminishing social esteem, injustice, and exposure to violence, bullying, or sexual harassment in the workplace, disproportionately affects nursing staff [4–6]. Therefore, it is imperative that healthcare institutions prioritize the provision of comprehensive support to nurses, the enhancement of their professional life quality, the affirmation of their right to decent work, and the promotion of overall well-being. These measures are vital not only to the individual welfare of nurses but also to the maintenance of high-quality patient care.

Perceived Organizational Support (POS) refers to the extent to which an organization values, respects, and cares for the well-being of its employees, in addition to the contributions and services they provide to the organization [7]. Manifestations of POS include the provision of a supportive work environment, access to training and development, and equitable compensation and rewards. Research indicates that substantial organizational support is inversely related to nurse burnout and turnover intentions [8], while positively associated with the enhancement of nurses’ psychological capital—crucial elements of their professional quality of life [9]. Consequently, this study posits a correlation between nurses’ perceived organizational support and their professional quality of life.

Beyond organizational support, the professional quality of life is fundamental to nurses’ discernment of decent work and overall well-being. Professional quality of life refers to the quality of life that helping professionals achieve through their work [10, 11]. This concept encompasses a range of positive and negative psychological impacts experienced while providing services to others [11]. Positive impacts, known as compassion satisfaction, refer to the pleasure derived from doing one’s work well, helping and caring for others, or from any effort made in one’s work [12]. Negative impacts, referred to as compassion fatigue, are negative effects experienced by helpers due to close contact with the suffering or trauma of others [12]. Over the past decade, this concept has been widely applied across various industries and has become an important perspective for studying professionals’ subjective experiences of their work [13]. Research has found that low levels of compassion satisfaction and high levels of compassion fatigue can lead to issues such as sleep disorders and physical/mental health in nurses [14]. Consequently, this research hypothesizes a positive relationship between nurses’ professional quality of life and their perception of decent work.

The United Nations’ Sustainable Development Goal 8, “Decent work and economic growth,” focuses on labor-related matters [15], aiming to foster sustained, inclusive, and sustainable economic growth by ensuring full and productive employment, alongside decent work for all [16]. The International Labor Organization (ILO) defines decent work as productive labor coupled with the protection of workers’ rights, fair income, adequate social protection, and employment opportunities [17]. Ultimately, the goal is to ensure that the vast majority of workers labor in conditions of freedom, justice, safety, and dignity. Duffy distill decent work to the micro-level, encompassing safe working conditions, hours that allow for free time and adequate rest, organizational values that complement family and social values, adequate compensation, and access to adequate healthcare [18]. Ferraro conceptualized decent work into seven domains: fundamental principles and values at work, adequate working time and workload, fulfilling and productive work, meaningful remuneration for the exercise of citizenship, social protection, opportunities, and health and safety [19]. Recent studies indicate that decent work correlates with heightened job satisfaction among nurses, diminished turnover intentions, and reduced burnout [20]. Accordingly, this research postulates a positive correlation between nurses’ perceptions of decent work and their well-being.

Well-being is a comprehensive and multifaceted concept, representing a positive mental state characterized by competence, emotional stability, engagement, meaning, optimism, positive emotions, positive relationships, resilience, self-esteem, and vitality [21, 22]. The American Nurses Association defines a healthy nurse as one who can maintain physical, intellectual, emotional, social, spiritual, personal, and professional well-being [23]. The critical role nurses play in patient care necessitates a focus on their professional well-being, which is inextricably linked to both their own health and patient outcomes. Empirical evidence suggests that a nurse’s professional well-being is a determinant of their work performance and caregiving capacity, with lower well-being associated with burnout, job dissatisfaction, and heightened turnover [24, 25]. Additionally, the concerning rates of nurse suicides highlight the urgency of prioritizing professional well-being within the nursing community [26].

**Fig. 1:**
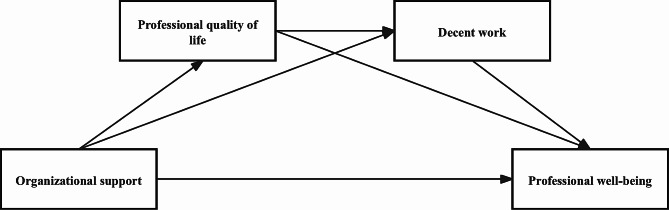
Hypothetical Model of the Mediating Role of Professional Quality of Life and Decent Work in the Relationship between Organizational Support and Professional Well-being among Nurses

The Organizational Support Theory (OST) underpins the study of the interplay among the constructs in question. It contends that the care and support an organization extends to its members are pivotal in fostering their loyalty and contributions [27]. Elevated levels of organizational support correlate with increased emotional attachment to, and a sense of responsibility towards, the organization; conversely, perceived deficiencies in support are associated with a higher propensity for employee turnover [28]. Organizational support mechanisms, such as salary increments and professional development opportunities, enhance employee engagement, job satisfaction, and, in turn, elevate the quality and integrity of work. Organizational support also promotes confidence, job satisfaction, and an overall state of well-being among employees [29]. Additionally, existing research indicates that higher levels of organizational support are associated with better professional quality of life among nurses [9], and there is a positive correlation between organizational support and nurses’ sense of well-being [30]. There is also a direct link between professional quality of life and nurses’ sense of well-being [31]. Furthermore, decent working conditions, such as fair compensation, reasonable workload, and a safe work environment, are typically associated with high levels of organizational support [32].Thus, according to OST, this study hypothesizes that employees’ perceptions of organizational support are posited to exert a direct effect on their well-being, with professional quality of life and perceptions of decent work acting as mediatory factors within this relationship (See Fig. 1).

In summary, although past research has explored the impact of organizational support on well-being, it has predominantly focused on job stress and mental health issues, without sufficiently delving into the dimensions of professional quality of life and decent work. Furthermore, current studies have relatively overlooked how organizational support can enhance professional quality of life and the perception of decent work, as well as how these variables further influence nurses’ professional well-being [9, 30–33]. Therefore, this study aims to fill this gap by utilizing the OST model to examine the direct and indirect relationships between organizational support and nurses’ well-being. This approach is intended to improve job satisfaction, reduce staff turnover rates, and ultimately enhance the quality of patient care.

## Methods

### Design

This study used a cross-sectional survey design, conducted in January 2024, and the research report adhered to the Strengthening the Reporting of Observational Studies in Epidemiology (STROBE) checklist [34].

### Participants recruitment

This study used convenience sampling to select registered nurses from five tertiary Grade A hospitals in Shanxi Province. These hospitals are located in the same city and share similarities in scale, management mode, nurse income levels, and welfare benefits. The research team collaborated with the leadership departments of the hospitals to select eligible participants from the member lists in the hospital management system. The research assistants then sent the electronic link to the participants. Additionally, to ensure the smooth recruitment process, we established communication and feedback mechanisms. We regularly communicated with the hospital research assistants to understand the recruitment progress and any issues encountered, making timely adjustments and improvements as needed.

### Inclusion and exclusion criteria

Based on previous research and discussions among team members, the inclusion criteria for nurses in this study are: (1) Registered nurses; (2) Having one year or more of work experience; (3) Voluntary participation in this study.

Exclusion criteria: (1) History of mental or psychological disorders; (2) Nurses in administrative or logistical positions(responsible for tasks such as patient meals, equipment sterilization, and patient transport).

### Sample size

The required sample size was determined via G*Power analysis (version 3.1), assuming a multiple linear regression, an alpha of 0.05, and a power of 0.95. Considering 20 variables—including 5 sociodemographic and 15 scale-related dimensions—the minimum sample size was calculated to be 222 nurses. To allow for a 10% margin of invalid questionnaires, the sample size was adjusted to a minimum of 244 nurses.

In this study, 842 questionnaires were initially collected. After excluding 50 questionnaires that did not meet the criteria, a total of 792 valid questionnaires were retained, resulting in an effective response rate of 94.06%.

### Instruments

#### Demographic characteristics

We used a custom demographic survey to collect basic information from nurses, including gender, age, education background, years of working, and professional title.

Perceived organizational Support Scale.

#### Perceived Organizational Support Scale (POSS)

This study employed the Chinese version of the Perceived Organizational Support Scale to measure nurses’ perception of organizational support. The scale, originally developed by Eisenberger [27] and later revised by McMillint, was further refined by Chinese scholar Hongmei Zuo [35], resulting in a new Chinese version that was validated among the nursing population and demonstrated good applicability. The scale comprises two dimensions: affective support (items 1 to 10) and instrumental support (items 11 to 13), totaling 13 items. Respondents rated each item on a 5-point Likert scale, ranging from “strongly disagree” to “strongly agree,” scored from 1 to 5, respectively. The total score ranges from 13 to 65, with higher scores indicating better perceived organizational support. In this study, the Cronbach’s α coefficient for the scale was calculated as 0.986. The Cronbach’s α for the dimensions were: affective support 0.984, instrumental support 0.962.

#### Professional Quality of Life Scale (Pro-QOLS)

The Chinese version of the Professional Quality of Life Scale was utilized in this study to assess the quality of professional life among nurses. The scale, originally developed by Stamm [11], underwent translation and revision by Chinese scholars [36], demonstrating good reliability and validity. Comprising three subscales: compassion satisfaction, burnout, and secondary traumatic stress, each with 10 items, the scale totals 30 items. Responses were rated on a 5-point Likert scale ranging from “never” to “very frequently,” with scores ranging from 1 to 5. In this study, for consistency, scores for compassion satisfaction were reverse-coded. Higher scores on the scale indicate higher levels of burnout and secondary traumatic stress, as well as lower levels of compassion satisfaction, reflecting lower quality of professional life. The Cronbach’s α coefficient for the scale in this study was calculated as 0.811. The Cronbach’s α for the dimensions were: compassion satisfaction 0.951, secondary traumatic stress 0.865, burnout 0.788.

#### The Decent Work Perceptions Scale (DWPS)

The Decent Work Perceptions Scale was utilized in this study to assess the level of perceived decent work among nurses. The scale developed by Chinese scholars has been widely used among nurses [20, 37]. It comprises five dimensions: work rewards (4 items), work position (3 items), career development (3 items), work recognition (3 items), and work atmosphere (3 items), totaling 16 items. Responses were rated on a 5-point Likert scale ranging from “completely disagree” to “completely agree,” with scores ranging from 1 to 5. The total score ranged from 16 to 80, with higher scores indicating stronger perception of decent work among nurses. In this study, the Cronbach’s α coefficient for the scale was calculated as 0.960. The Cronbach’s α for the dimensions were: work rewards 0.916, work position 0.837, career development 0.859, work recognition 0.946, and work atmosphere 0.899.

#### Nurse Occupational Well-being Questionnaire (NOWQ)

The Nurse Occupational Well-being Questionnaire, developed by Ling Chen a [38], was used in this study to assess nurses’ well-being. The questionnaire comprises five dimensions: “Welfare Treatment,” including 4 items (Items 1, 6, 11, 16); “Interpersonal Relationships,” including 4 items (Items 2, 7, 12, 18); “Work Values,” including 5 items (Items 3, 8, 13, 17, 19); “Management,” including 3 items (Items 4, 9, 14); and “Job Characteristics,” including 3 items (Items 5, 10, 15), totaling 19 items. Responses were rated on a 6-point Likert scale, ranging from 1 (“strongly disagree”) to 6 (“strongly agree”). The total score ranged from 19 to 114, with higher scores indicating higher work well-being among nurses. In this study, the Cronbach’s alpha coefficient for the scale was calculated as 0.967, indicating high internal consistency. The Cronbach’s α for the dimensions were: welfare treatment 0.937, interpersonal relationships 0.922, work values 0.928, management 0.941, and job characteristics 0.810.

### Data collection

In this study, all questionnaires were converted into an electronic format using ‘Wen Juan Xing’ and a corresponding link was generated (www.wjx.cn). Subsequently, the researchers provided standardized training to the research assistants. The research assistants were responsible for briefly introducing the study’s purpose and procedures to the nurses, ensuring they understood the study’s objectives and methods. The research assistants then sent the electronic link to all eligible registered nurses through their human resources management system. Nurses could start answering the questionnaire by clicking the link. Responses were automatically compiled into a spreadsheet for uniformity.

### Informed consent

Participating nurses were thoroughly informed about the purpose of the study, the method of completing the questionnaires, the guarantee of anonymity, and the principles of voluntary participation. Nurses were assured they could withdraw without consequence at any time. The informed consent process was conducted online, where nurses were provided with detailed information about the study and asked to click a voluntary participation button to proceed to the questionnaire. If they chose to decline, the session ended immediately. Nurses’ decisions to complete or decline the questionnaire were confidential and known only to the individual nurse, with no external pressure.

### Data management

All data in this study were kept confidential and securely stored in accordance with research ethics involving human participants. No identifying information was stored; only basic demographic data were collected. After data collection, the data were stored in secure electronic folders, managed by authorized researchers, and only accessible to the researchers of this study.

### Statistical analysis

Data were analyzed using SPSS version 27.0 (IBM Corp., Armonk, NY, USA) and the PROCESS Macro. Descriptive statistics summarized the demographic details of the nurse participants and the scores of measured variables. Pearson correlation analysis was employed to examine the relationships between variables. Mediation analysis was conducted using PROCESS version 4.0, with Bootstrap resampling based on 5,000 iterations. The significance of path coefficients was evaluated with a 95% confidence interval. The significance of the indirect effects was determined by examining whether the 95% confidence intervals included zero. If zero was not included, the indirect effect was considered statistically significant. In this study *p*-values less than 0.05 were considered to indicate statistical significance. To evaluate data validity and control for potential bias arising from the use of extensive questionnaire items, this study applied Harman’s single-factor test. The analysis identified 8 factors with eigenvalues greater than one. However, the first factor accounted for only 46.64% of the variance, which is below the commonly accepted critical threshold of 50% [39]. This indicates that no single factor dominated the variance, suggesting that the observed variance is distributed across multiple factors rather than being attributed to a single common method. Consequently, common method bias did not significantly impact the study. Additionally, the remaining factors accounted for smaller portions of the variance (9.18%, 6.10% etc.), further supporting the robustness of the data against common method bias. These findings affirm that the study’s results are reliable and not significantly influenced by the measurement method employed.

## Results

### Participant characteristics

Table 1 displays the demographic characteristics of the participants. The data reveals that the mean age of nurses was 33.75 ± 7.07. Moreover, the majority of surveyed nurses were female (95.7%). In terms of education level, most nurses held a bachelor’s degree (88.4%). Regarding years of working experience, the largest proportion of nurses (58%) had been working for 5 to 15 years. The predominant professional title among the surveyed nurses was supervisor nurse(indicating a nurse with extensive nursing experience and advanced professional skills) (47.1%).

**Table 1:** Demographic characteristics of participants

Variable	Category	Frequency	Percentage(%)
Gender	Male	34	4.3
	Female	758	95.7
Education Level	Below College	73	9.2
	Bachelor’s	700	88.4
	Master’s and Above	19	2.4
Years of Working	< 5	190	24
	5–15	459	58
	16–25	79	10
	> 25	64	8.1
Professional Title	Nurse	98	12.4
	Senior nurse	261	33
	Supervisor nurse	373	47.1
	Associate chief nurse and Above	60	7.6

### The scores of organizational supports, professional quality of life, decent work, and well-being

In this study, nurses reported a mean score of 44.29 ± 13.48 for perceived organizational support and 52.46 ± 14.05 for perceived decent work. The average score for professional quality of life was 84.97 ± 12.64, while the mean score for well-being was 82.23 ± 18.36. Detailed scores for each dimension are showed in Table S1-S4.

### Correlation analysis

The correlation analysis revealed a negative correlation between perceived organizational support and professional quality of life (*r* = -0.318, *p* < 0.001). In contrast, perceived organizational support was positively correlated with perceptions of decent work (*r* = 0.867, *p* < 0.001) and well-being (*r* = 0.829, *p* < 0.001). There was a negative correlation between professional quality of life and both perceived decent work (*r* = -0.358, *p* < 0.001) and well-being (*r* = -0.380, *p* < 0.001). Additionally, decent work was positively correlated with well-being (*r* = 0.888, *p* < 0.001) (See Table 2). In addition, the results of this study show that compassion satisfaction is negatively correlated with all dimensions of decent work, while burnout and secondary traumatic stress are positively correlated with these dimensions. Detailed results are shown in Table S5.

**Table 2:** Correlations among organizational support, professional quality of life decent work and well-being (*N* = 792)

Variables	Organizational Support	Professional quality of life	Decent Work	Well-being
Organizational Support	1			
Professional quality of life	-0.318**	1		
Decent Work	0.867**	-0.358**	1	
Well-being	0.829**	-0.380**	0.888**	1

**p < 0.001

### Mediated effect analysis

**Fig. 2:**
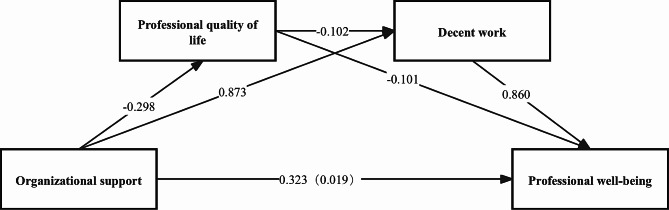
Chain Mediation Model of Organizational Support, Professional Quality of Life, Decent Work, and Well-Being Among Nurses

As shown in Fig. 2, the results of this study indicate that organizational support has a direct negative impact on professional quality of life (β= -0.298, *p* < 0.001), while it has direct positive effects on decent work (β = 0. 873, *p* < 0.001) and well-being (β = 0.323, *p* < 0.001). Professional quality of life exerts direct negative influences on decent work (β= -0.102, *p* < 0.001) and well-being (β= -0.101, *p* < 0.001). Furthermore, decent work demonstrates a direct positive effect on well-being (β = 0.860, *p* < 0.001). Mediation analysis reveals that both professional quality of life (*β* = 0.022, BootLLCI = 0.011, BootULCI = 0.035) and decent work (*β* = 0.551, BootLLCI = 0.489, BootULCI = 0.614) serve as mediators between organizational support and well-being. Moreover, the serial mediation effect of organizational support → professional quality of life → decent work → well-being is significant (*β* = 0.019, BootLLCI = 0.010, BootULCI = 0.030). The mediation effects account for 52.39% of the total effects. (See Table 3)

**Table 3:** Analysis of regression relationships of variables

Effect	Item	β	BootSE	BootLLCI	BootULCL
Direct effects	OS→PQL	-0.298	0.037	-0.370	-0.227
	OS→DW	0.873	0.022	0.830	0.918
	OS→ WB	0.323	0.055	0.217	0.433
	PQL→ WB	-0.101	0.024	-0.147	-0.056
	DW→ WB	0.860	0.045	0.771	0.948
	PQL→DW	-0.102	0.022	-0.145	-0.059
Total effect	OS→PQL	1.130	0.027	1.077	1.183
Indirect effects	OS→PQL→WB	0.022	0.006	0.011	0.035
	OS→DW→WB	0.551	0.032	0.489	0.614
	OS→PQL→ DW→ WB	0.019	0.005	0.010	0.030
	Total	0.593	0.032	0.531	0.656

Abbreviations: OS, organizational support; PQL, professional quality of life; DW, decent work; WB, well-being; β, standardized regression coefficient; SE, standard error; BootLLCI, bootstrapping lower limit confidence interval; BootULCI, bootstrapping upper limit confidence interval

## Discussions

This study aimed to explore the relationships among organizational support, professional quality of life, decent work, and well-being among nurses. The survey results indicated a direct correlation between organizational support and nurses’ well-being, and establish that professional quality of life affects nurses’ perceptions of decent work, which in turn impacts their well-being. Moreover, professional quality of life and decent work serve as mediators between organizational support and well-being.

The findings of this study indicate that organizational support significantly influences nurses’ professional well-being, aligning with previous research [40]. In the context of the nursing profession, where job pressures and long hours are prevalent, the importance of organizational support is particularly emphasized [41]. Supportive perceptions within the workplace contribute to nurses’ positive work attitudes, which in turn boost work efficiency and well-being [30]. Hence, it is imperative for healthcare institutions to focus on augmenting the perception of organizational support by fostering appropriate work conditions, benefits, and career growth opportunities, thereby reinforcing nurses’ sense of being valued and supported.

The findings of this research highlight the pivotal role that professional quality of life plays in enhancing perceptions of decent work among nurses. It has been observed that nurses with a high professional quality of life report greater levels of decent work. This relationship is critically important in the context of the current labor market, characterized by changing work dynamics, intensified job competition, and the integration of artificial intelligence. The emphasis on improving professional quality of life is increasingly recognized by management as essential for organizational success [42, 43]. Nurses with higher professional quality of life typically experience lower burnout and secondary traumatic stress, and they maintain higher compassion satisfaction. This equips them with the resilience needed to manage job stress and to meet challenges positively, which in turn helps reduce burnout and traumatic stress [44, 45]. Additionally, their elevated compassion satisfaction—a reflection of the fulfillment they gain from caring for others—acts as a motivator, spurring them to engage more fully in their roles [46]. As a result, nurses with high professional quality of life tend to perform tasks more effectively and provide superior care, which contributes to higher standards of decent work.

Furthermore, the study findings demonstrated a direct impact of decent work on nurses’ well-being, aligning with previous research findings [47]. Decent work, an inclusive term, involves facets such as suitable working conditions, equitable treatment, opportunities for career progression, and upholding personal dignity [48]. Concerning the work environment, decent work necessitates the provision of a safe, healthy, and harmonious workplace [49]. In such an environment, nurses are better equipped to fulfill their duties, thereby reducing the physical and mental stress caused by unfavorable working conditions. Additionally, decent work emphasizes fair treatment and opportunities for career development [50]. This implies that nurses should receive compensation and benefits commensurate with their contributions, along with fair opportunities for career advancement and skill development. Such treatment not only meets nurses’ material needs but also enhances their sense of well-being. Conversely, when nurses are unfairly treated or unjustly evaluated, their work motivation and job satisfaction can be severely impacted [51], thereby affecting their professional well-being. Finally, decent work also underscores personal dignity and respect [52]. Respect in the workplace, including the appreciation of nurses’ perspectives and efforts, is vital for their overall well-being.

Finally, the study results indicate that professional quality of life and decent work act as mediators between organizational support and well-being, consistent with the assumptions of the Organizational Support Theory [53], thereby constructing a chain-mediated model. Organizational support has a direct positive effect on nurses’ well-being and an indirect effect via the mediating variables of professional quality of life and decent work. As an essential asset, organizational support equips nurses with the psychological backing and job stability necessary for an enriched work life and quality of life [54]. Enhanced professional quality of life contributes to a heightened perception of decent work, characterized by substantial material benefits and intangible assets such as respect, achievement, and self-esteem in the work setting [55]. As nurses perceive improvements in their professional quality of life, they tend to thrive in their roles, gain acknowledgment, and solidify their perception of decent work, fostering a beneficial cycle [11]. Well-being reflects a holistic psychological state derived from life and work satisfaction. In the nursing profession, the well-being drawn from respect, achievement, and self-worth associated with decent work is particularly impactful. Hence, a cyclical synergy exists among organizational support, professional quality of life, and decent work, each amplifying nurses’ well-being. Organizational support, as the nexus, fosters nurses’ well-being by enhancing their professional quality of life and perception of decent work.

The findings of this study hold significant implications for nursing practice, organizational management, and policy development within healthcare institutions. First, it is imperative that healthcare institutions elevate the organizational support provided to nurses by ensuring adequate resources, support mechanisms, and nurturing work environments. For example, the implementation of employee assistance programs offering psychological support and counseling services can assist nurses in managing occupational stress and emotional burdens [56]. Second, the enhancement of nurses’ professional quality of life should be a focal point [45], which includes mitigating nurse fatigue and secondary traumatic stress, as well as fostering compassion satisfaction. Adopting flexible scheduling and ensuring adequate rest and vacation time can help reduce work pressure and fatigue. Further, promoting team-building initiatives and professional development can augment nurses’ sense of teamwork and their competencies, increasing job satisfaction and compassion [57]. Additionally, organizations should adopt policies and practices to promote decent work for nurses. Healthcare institutions might, for instance, introduce equitable salary structures to reflect nurses’ professional input and offer bonuses and benefits as performance incentives [58]. Lastly, nurses should have access to comprehensive career development paths that include educational programs, opportunities for academic growth, and advancement prospects, thereby continuously enhancing their professional skills and career trajectories [59]. Such strategic measures are not only beneficial for the nurses but also for the patients and the healthcare system as a whole, contributing to a more effective and compassionate care environment.

### Limitation

This study has a few limitations. Firstly, it used a cross-sectional survey design, which limits our understanding of causal relationships among variables [60]. Future research could use longitudinal designs to explore causal relationships more effectively. Secondly, the use of self-report questionnaires may lead to social desirability bias, with participants potentially offering responses they deem socially acceptable [61], thus compromising result objectivity. To mitigate this, subsequent studies might incorporate mixed methods, including qualitative interviews, to enrich data validity. Lastly, the study had a small sample size of male nurses and fewer experienced nurses, which could impact the generalizability of findings [62]. Future research efforts should aim for larger and more diverse samples, encompassing a broader range of nurses in terms of gender, age, and professional experience, to enhance the representativeness of the data.

## Conclusions

In conclusion, this study has examined the impact of organizational support on nurses’ professional well-being. By analyzing the data, we found that organizational support has a direct effect on nurses’ professional well-being. Professional quality of life and decent work act as mediatory factors in this relationship. Additionally, the findings suggest that healthcare institutions should prioritize organizational support measures to enhance nurses’ professional quality of life, perceptions of decent work, and professional well-being. Future research should continue to explore the mechanisms through which organizational support influences various aspects of professional well-being and identify additional mediatory factors that may play a role. This study could provide valuable insights for nursing policymakers and administrators, enabling the development of more effective strategies to enhance nurses’ professional well-being.

### Electronic supplementary material

Below is the link to the electronic supplementary material.

Supplementary Material 1

## Data Availability

The data that support the findings of this study are available from the corresponding author on reasonable request.

## Abbreviations

COVID-19: Corona Virus Disease 2019
STROBE: Strengthening the Reporting of Observational studies in Epidemiology
ILO: International Labor Organization
SPSS: Statistical Product and Service Solutions
OST: Organizational Support Theory
POSS: Perceived Organizational Support Scale
DWPS: The Decent Work Perception Scale
Pro-QOLS: Professional Quality of Life Scale
NOWQ: Nurse Occupational Well-being Questionnaire
